# Gut Microbial Antigenic Mimicry in Autoimmunity

**DOI:** 10.3389/fimmu.2022.873607

**Published:** 2022-04-27

**Authors:** Nahir Garabatos, Pere Santamaria

**Affiliations:** ^1^Institut D’Investigacions Biomèdiques August Pi i Sunyer, Barcelona, Spain; ^2^Julia McFarlane Diabetes Research Centre (JMDRC), Snyder Institute for Chronic Diseases, Cumming School of Medicine, University of Calgary, Calgary, AB, Canada; ^3^Department of Microbiology, Immunology and Infectious Diseases, Snyder Institute for Chronic Diseases, Cumming School of Medicine, University of Calgary, Calgary, AB, Canada

**Keywords:** gut microbiota, dysbiosis, autoreactive T-cell responses, autoimmune disease, molecular mimicry, gut microbial metabolites, immunoregulation, gut microbial homeostasis

## Abstract

The gut microbiota plays a major role in the developmental biology and homeostasis of cells belonging to the adaptive and innate arms of the immune system. Alterations in its composition, which are known to be regulated by both genetic and environmental factors, can either promote or suppress the pathogenic processes underlying the development of various autoimmune diseases, including inflammatory bowel disease, multiple sclerosis, systemic lupus erythematosus, type 1 diabetes and rheumatoid arthritis, to just name a few. Cross-recognition of gut microbial antigens by autoreactive T cells as well as gut microbe-driven alterations in the activation and homeostasis of effector and regulatory T cells have been implicated in this process. Here, we summarize our current understanding of the positive and negative associations between alterations in the composition of the gut microbiota and the development of various autoimmune disorders, with a special emphasis on antigenic mimicry.

## Introduction

Our natural anatomic barriers, including the skin and mucous membranes, are colonized by billions of microorganisms that live in a symbiotic relationship with the host. The gut microbiota, for example, is composed of different species of commensal bacteria, fungi, viruses and archaeas. During natural evolution, the host and the commensal microorganisms that colonize it have co-evolved to develop complex relationships that impact numerous host biological processes, including immune system homeostasis. Multiple gut microbial species, dietary compounds and/or microbial metabolites contribute to these processes. Gut dysbiosis or disruption of the gut barrier function can trigger a loss of tolerance to gut microbial antigens, eliciting immune responses that can potentially promote not only local inflammation, but also distal autoimmune phenomena. Here, we review our current understanding of the positive and negative associations between alterations in the composition of the gut microbiota and autoimmunity, including known examples of antigenic mimicry. Altogether, this information paints a complex landscape that exposes knowledge gaps and research opportunities.

## Gut Microbiota and Homeostasis of the Gut-Associated Immune System

The crosstalk between the immune system and the gut microbiome begins in the immediate postnatal period. The host immune system matures during the first few years of life in a dynamic relationship with the gut microbiome, leading to a state of equilibrium at around 3 years of age (1, 2). The largest microbial colonization of the gut occurs during and immediately after birth (3), and is impacted by factors such as the delivery mode (4) and breast feeding (5, 6). It has been recently shown that a weaning reaction to the microbiota, leading to the generation of RORgamma(+) Treg cells *via* bacterial and dietary metabolites, including short-chain fatty acids (SCFAs) and retinoic acid, is required for resistance to immunopathologies in the adult, such as colitis and allergic inflammation (7). Related to this, intestinal secretion of the antimicrobial peptide cathelicidin upon exposure to commensal bacteria has been reported to shape a protective neonatal gut microbiota against pancreatic autoimmunity (8).

Maintenance of immune tolerance against the gut microbiota is regulated by complex processes that are orchestrated in the gut-associated lymphoid tissue (GALT). GALT-associated innate immune cells can distinguish between potentially pathogenic microbial components and their harmless commensal counterparts by recognizing pathogen-associated pattern recognition receptors (PRRs), which ultimately lead to the activation of antigen-specific CD4+ and CD8+ T cell effectors. In the healthy steady state, the GALT-associated B cells produce gut microbial antigen-specific IgAs to suppress mucosal penetration by commensals, hence the induction of potentially harmful local immune responses by effector T cells (9). This process is supported by both dendritic cells and T-follicular helper (TFH) cells. In addition, Peyer´s patch-associated Th17 cells promote Ig class switching and production of soluble IgA *via* IL-21 (10). Local induced FoxP3+ Treg cells (iTregs) also contribute to the maintenance of normal immune homeostasis, by both suppressing pathogenic effector T cell responses and promoting IgA production (11). Interestingly, there is evidence suggesting that most of the TFH cells in the Peyer’s patches arise from pre-existing Treg cells and Th17-type cells (12, 13).

**Figure 1:**
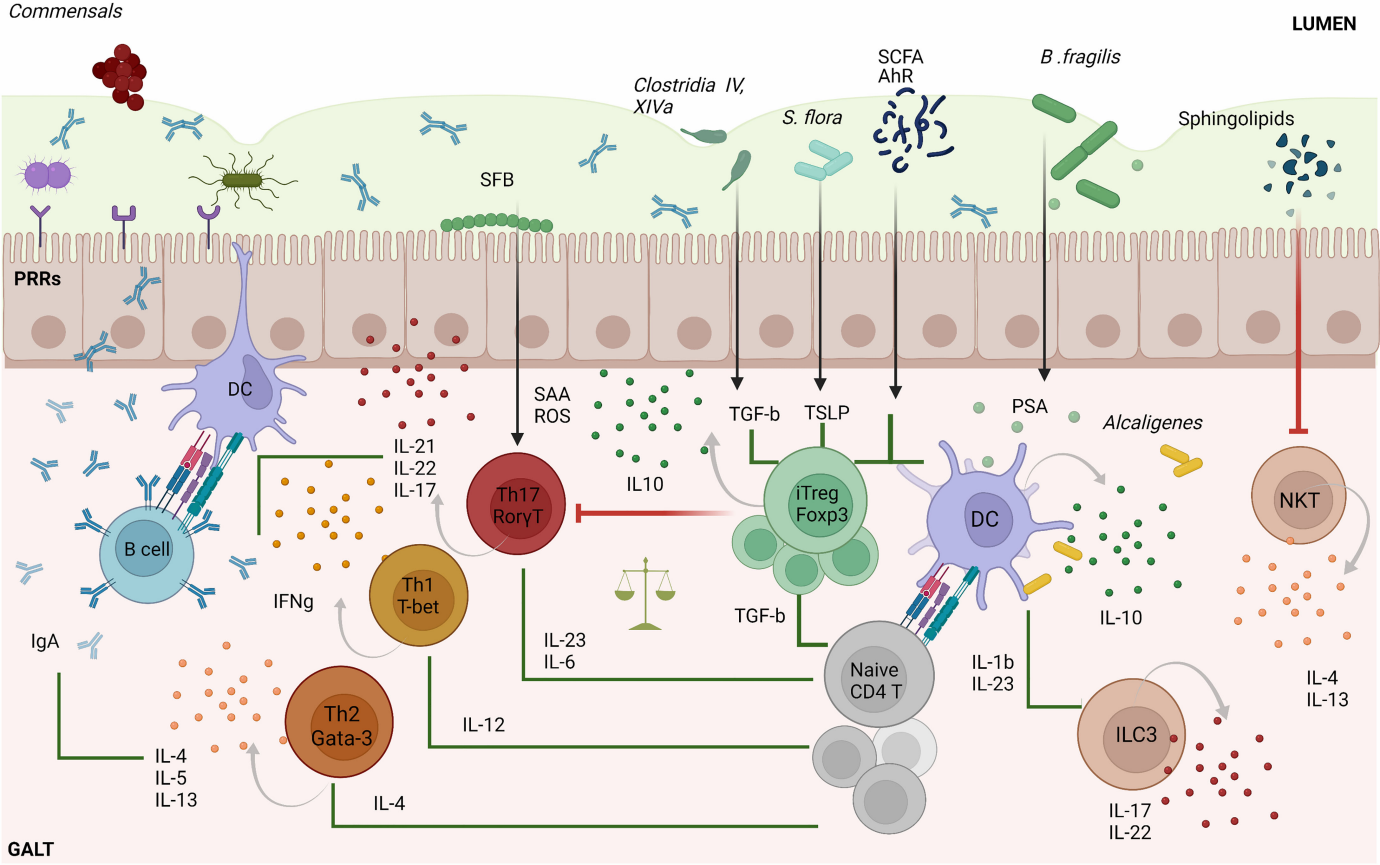
Microbiota–T cell crosstalk in the maintenance of gut homeostasis. Commensal bacteria can trigger pattern recognition receptors (PPRs) on enterocytes and/or activate antigen specific CD4+T cell responses *via* dendritic cells (DC). Naïve CD4+ T cells can differentiate into four major cell types: Th1, Th2, Th17 and Tregs. The differentiation of each Th type requires specific transcription factors and cytokine sets, as shown in the figure. Th1 cells play an important role in eliminating intracellular pathogens while Th2 control parasitic infections and extracellular pathogens trough the induction of antibody responses. The primary role of Th17 cells is to control infection, but also contributes to intestinal homeostasis by inducing protective IgA responses. SFB commensal bacteria promote gut Th17 cell responses by triggering the intestinal production of SAA and ROS. iTreg cells play a key role in controlling Th cell responses and in maintaining gut immune homeostasis. Several commensal bacteria such as *Clostridia* spp., dietary compounds (SCFA) and AhR ligands participate in the maintenance of tolerance by inducing gut Treg cell responses or by imprinting tolerogenic features on DCs, as is the case for *Alcaligenes* spp. Other immune cell types such as invariant natural killer T-cells (iNKT) are suppressed and controlled by bacterial sphingolipids preventing intestinal pro-inflammatory responses. In addition, type 3 innate lymphoid cells (ILC3) promote protective Th17 responses *via* IL-22 and IL-17. The types of bacteria implicated in particular T cell differentiation pathways as well as metabolites are indicated in the figure. SFB, segmented filamentous bacteria; AhR, Aryl hydrocarbon receptor; TGF-β, transforming growth factor-beta; SCFA, short-chain fatty acids; PSA, polysaccharide A; SAA, serum amyloid A protein; ROS, reactive oxygen species; GALT, gut-associated lymphoid tissue; TSLP, thymic stromal lymphopoietin; iTreg, induced regulatory T cell.

Commensal bacteria and metabolites play an active role in the development and regulation of adaptive immune responses in the gut (**Figure 1**). In mice, segmented filamentous bacteria (SFB) living in the small intestine help promote the induction of protective, pathogen-specific Th17 responses (14, 15) by triggering the intestinal production of serum amyloid A protein (SAA) and reactive oxygen species (ROS) (16). In humans, the *Bifidobacterium B. adolescentis* might play a similar role (17). Other commensals promote the activation of Th17-suppressing Treg cell responses by eliciting the production of intestinal thymic stromal lymphopoietin (TSLP) (18). Gut bacteria can also modulate Th1 responses to promote gut microbial tolerance. In mice, for example, gut microbes can suppress local Th1 cell responses *via* CX3CR1+ mononuclear phagocytes to favor a local tolerant state (19). In contrast, when *Klebsiella*, which is normally found in the oral cavity, ectopically colonizes the gut, it activates CD11b^–^CD103^+^ dendritic cells (DCs), promoting the activation of pro-inflammatory Th1 cell responses (20).

Other gut bacteria contribute to this process by inducing local Treg cell responses. *Clostridium* clusters IV and XIVa stimulate the secretion of transforming growth factor (TGF)-β by intestinal epithelial cells, promoting the differentiation and expansion of Treg cells in the colonic lamina propria (21). Likewise, *F. prausnitzii* induces the formation of T-regulatory type 1 (TR1)-like cells *via* TLR4-mediated activation of DCs (22). Moreover, *R. hominis* has been associated with activation of FoxP3+ Treg cells in the lamina propria (23). Commensal microbial metabolites also contribute to promoting local Treg cell responses. *Eubacterium* spp. produce SCFAs, mainly butyrate, that contribute to local immune homeostasis *via* several mechanisms (24–29). Microbial polysaccharides (PS) have also been associated with this process (30). In mice, for example, *B. fragilis* promotes the formation of tolerogenic CD103^+^ DCs and IL-10-producing FoxP3^+^ Treg cells *via* PSA-TLR2 signaling (31, 32).

Maintenance of gut microbial tolerance also involves the induction and regulation of other types of T cell responses. Recently, 11 bacterial strains were identified in healthy humans that induce protective IFNγ-producing CD8+ T cell responses in the intestine (33). In mice, invariant natural killer T-cells (iNKT), which bridge the innate and adaptive immune systems, have also been shown to be regulated by the host microbiota. *B. fragilis* sphingolipids, for example, modulate host colonic iNKT cell homeostasis, promoting gut barrier integrity (34).

Although most gut microbes reside in the lumen or on the gut epithelium, some commensal bacteria such as *Alcaligenes* spp., *Achromobacter* spp., *Bordetella* spp. and *Ochrobactrum* spp, exist in lymphoid follicles, Peyer’s patches and mesenteric lymph nodes of both healthy mice and humans (35). These bacteria trigger local interleukin-10 (IL-10) and IL-22 production from DCs and Type 3 Innate Lymphoid cells (ILC3), respectively. Whereas IL-10 suppresses the development of pro-inflammatory Th17 responses against commensals, IL-22 signaling favors bacterial colonization of lymphoid tissues.

Thus, the microbiota and the host immune system co-exist in a unique symbiotic relationship, where the host fosters gut colonization by microbes that are beneficial to the host, and/or help it suppress immune responses against these commensals.

## Disruption of Microbial Homeostasis Versus Pathogenic Immunity

**Figure 2:**
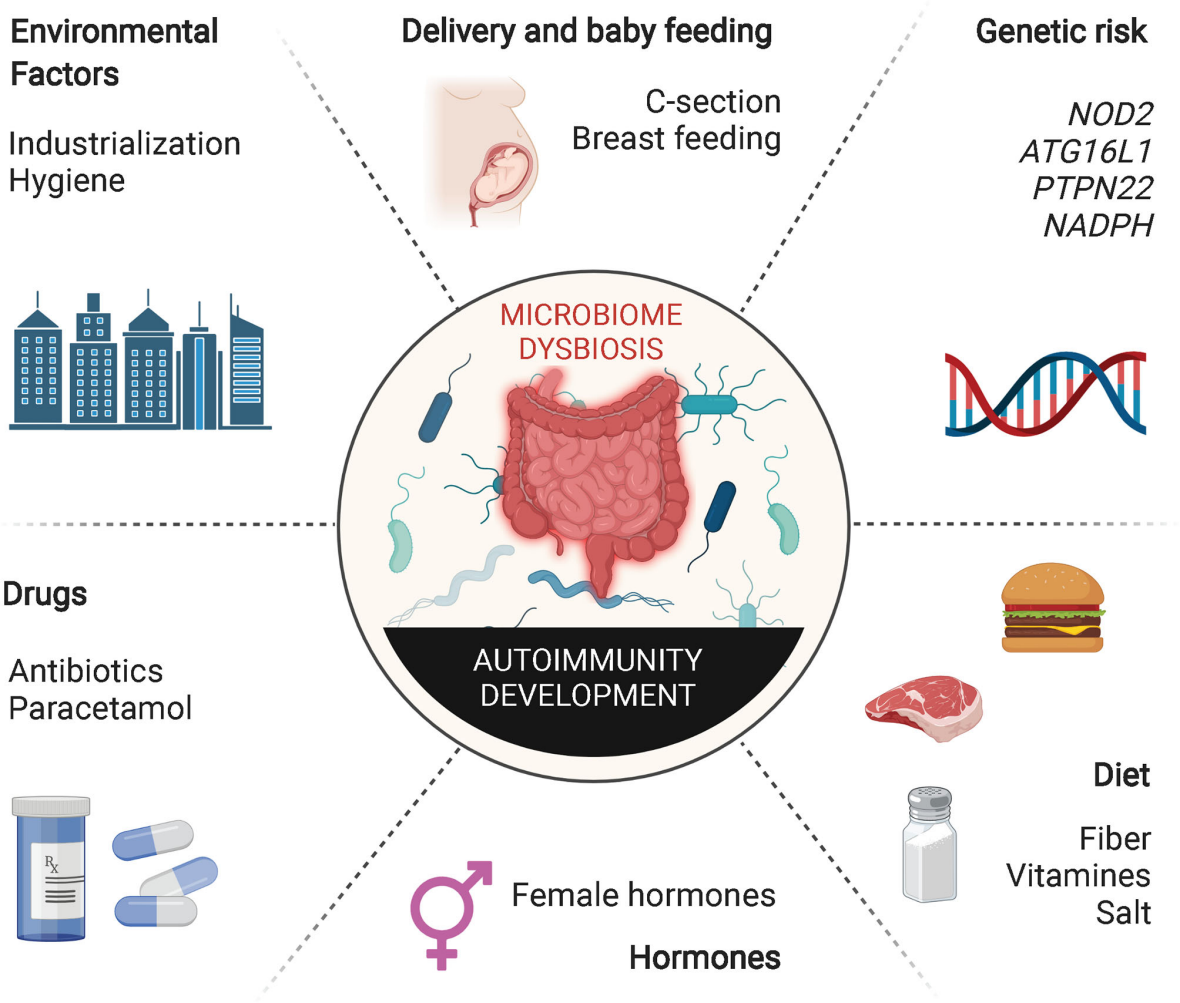
Extrinsic and intrinsic factors inducing gut dysbiosis. Host genetic susceptibility and hormones as well as various host-extrinsic factors such as intake of specific drugs, unhealthy diets, inappropriate microbial exposure, childbirth delivery or breast feeding may induce alterations in the composition of the gut microbiota. Decreased richness and perturbations in taxonomic commensal and metabolite composition have been extensively associated with the development of multiple autoimmune inflammatory disorders.

Dysregulation of tolerance to gut microbes can lead to the development of intestinal inflammatory processes, such as Crohn’s disease (CD) and ulcerative colitis (UC). Changes in the lifestyle of individuals living in industrialized societies during the last century have transformed how humans are exposed to environmental microbes. Excessive use of antibiotics, increase in hygiene, changes in childbirth mode and maternal breast-feeding patterns and poor nutritional habits have conspired with normal genetic determinants to increase the incidence of various immune-mediated diseases, betraying the beneficial role that these genetic determinants have on the host in the absence of these behavioral/societal changes (**Figure 2**). This putative association between decreased exposure to microbes and the rapid rise in the incidence and prevalence of chronic inflammatory disorders in industrialized societies has been conceptualized in the “hygiene hypothesis” (36). Various lines of experimental evidence in rodents support this hypothesis. Nonobese diabetic (NOD) mice as well as Biobreeding (BB) rats housed in conventional, non-specific pathogen-free (SPF) conditions develop a significantly decreased incidence of type 1 diabetes (T1D). In addition, infection of these rodent strains with various pathogens suppresses their autoimmune disease proclivity (37–39). Similar effects have been observed in systemic lupus erythematosus (SLE)-prone (NZB x NZW) F1 mice, where infection with *P. berghei* suppressed the development of lupus nephritis and prolonged survival (40), and in murine models of allergy, where microbial pathogen exposure suppresses disease. Although the precise mechanisms underlying these associations remain unclear, it has been suggested that excessive “hygiene” somehow interferes with adequate development of Treg cells.

Thus, antibiotics can have a profound impact on microbiome diversity and, as a result, on the host´s susceptibility to allergic and/or autoimmune diseases. In mice, antibiotics can increase the susceptibility of murine models to these pathological processes. In humans, the effects of antibiotic exposure on these disorders vary as a function of the timing of administration. Whereas excessive use of antibiotics during childhood may increase the susceptibility of children to atopic diseases, antibiotic use during adulthood may help suppress certain autoimmune disease processes (41). For example, excessive oral antibiotic use by mothers or newborns has been associated with increased susceptibility to T1D and asthma during childhood. In contrast, antibiotic-mediated resolution of *A. actinomycetemcomitans* infections (e.g. in periodontitis) in adults have been associated with suppression of rheumatoid arthritis (RA) (42), presumably due to the ability of the pore-forming toxin of this facultative anaerobe to promote protein citrullination, a major target of RA-associated autoantibodies.

Genetics and sex are additional key variables. Allelic variation at genes such as *NOD2*, encoding the intracellular PRR Nucleotide Binding Oligomerization Domain Containing 2, is strongly associated with susceptibility or resistance to inflammatory bowel disease (IBD). Microbial colonization of NOD male mice results in increased levels of serum testosterone and protection against T1D development (43, 44).

**Figure 3:**
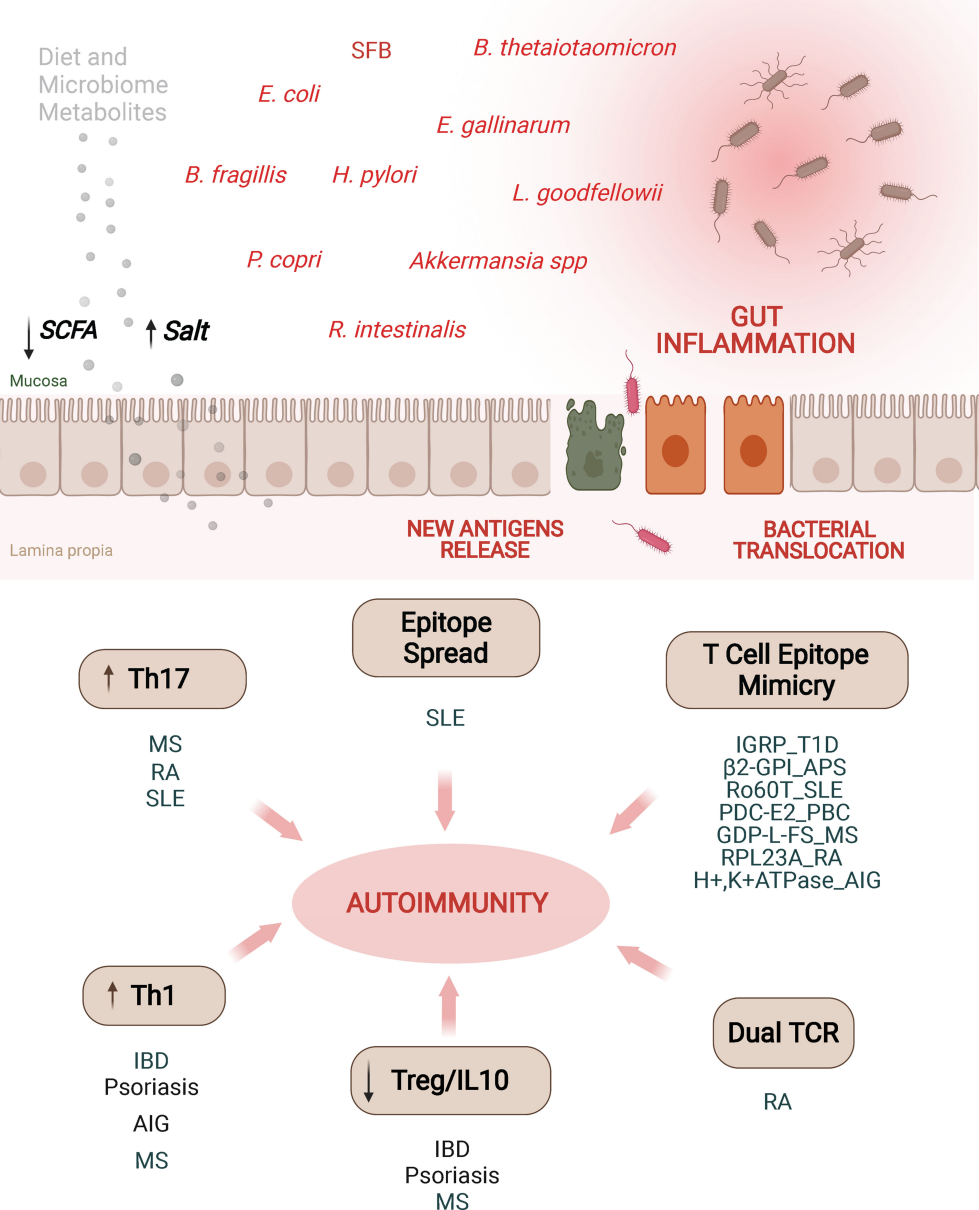
Alterations in the microbiota may promote autoimmunity through different mechanisms. Alterations of intestinal permeability caused by diet, bacterial metabolites, dysbiosis or pathobionts might increase exposure of gut microbial antigens to the gut associated lymphoid tissue. These adverse events have been associated with various autoimmune disorders through different mechanisms. Induction of Th17/Th1 cell responses, impaired or low levels of IL10-secreting Treg cell types, epitope spreading, dual TCR recognition or antigenic mimicry are some of the mechanisms. T1D, type 1 diabetes; AIG, autoimmune gastritis; IBD, inflammatory bowel disease; RA, rheumatoid arthritis; SLE, systemic lupus erythematosus; PBC, primary biliary cholangitis; MS, multiple sclerosis; SCFA, short chain fatty acids; MS, multiple sclerosis; β2-GPI, β2-glycoprotein I; APS, anti-phospholipid syndrome; PDC-E2, pyruvate dehydrogenase complex; GDP-L-FS, guanosine diphosphate-L-fucose synthase; RPL23A, arthritis-related autoantigen 60S ribosomal protein L23a; IGRP, islet-specific glucose-6-phosphatase catalytic subunit-related protein.

Despite all these important observations linking alterations in the gut microbiota with different autoimmune and allergic responses, the precise underlying mechanisms are not fully understood. Nevertheless, there is evidence suggesting that this is a multifactorial process, involving microbial-induced polarization of gut-associated T cells toward pathogenic subsets, bystander activation of autoreactive T cells, activation of T cells co-expressing dual (autoreactive and gut microbial antigen-specific) TCRs, and antigenic mimicry (**Figure 3**).

Gut dysbiosis or infections with pathobionts may disrupt gut immune homeostasis by polarizing T cell responses. Apoptosis during microbial infection drives autoreactive Th17 cell responses (45). Furthermore, there is evidence that commensals and gut pathogens can trigger differential cytokine expression patterns in gut T-helper cell subsets. Thus, whereas murine SFB promote the formation of IL-10-expressing (non-inflammatory) Th17 cells in the steady state, *C. rodentium* infection induces the formation of interferon-γ+ (pro-inflammatory) Th17 cells instead (46). In mice, another bacterium, *A. muciniphila*, promotes TFH cell formation under physiological conditions, but Th17 cell formation in the context of inflammation (47). In humans, *A. muciniphila*, which is enriched in the microbiota of patients with Multiple Sclerosis (MS), can skew differentiation of T cells into the Th1 cell subset *in vitro* (48, 49).

Antigenic cross-reactivity or molecular mimicry is another mechanism by which certain gut microbial antigens might be able to trigger T cell responses against autoantigens. Persistent colonization of the host with bacteria expressing cross-reactive epitopes in a host carrying high-risk Human Leukocyte Antigen (HLA) genes might trigger the sustained activation of cross-reactive autoreactive T cells in the gut, particularly if there is a loss in gut barrier integrity. Various autoantigen orthologues and non-orthologous mimotopes of autoantigens encoded in the microbiota have been implicated in the activation of autoreactive T cell responses in various autoimmune disorders (**Table 1**). Ro60-specific CD4+ T cell hybridomas (targeting the Sjögren’s syndrome Antigen A (SSA)), have been shown to cross-react, in HLA-DR3 transgenic mice, with an orthologous antigen expressed by *Capnocytphaga ochracea* (52). Likewise, DRB1*04:01-restricted T cells targeting β2-glycoprotein I (β2GPI) epitopes in patients with anti-phospholipid syndrome (APS) have been shown to cross-react with a bacterial peptide from *R. intestinalis*, inducing pro-inflammatory Th1 cell responses *in vitro* (51). Another example of molecular mimicry involves the rheumatoid arthritis (RA)-relevant autoantigens N-acetylglucosamine-6-sulfatase (GNS) and filamin A (FLNA). Multiple gut microbial peptide epitopes are structural mimics of these synovial proteins (50). Likewise, the murine diabetogenic IGRP_206-214_ epitope is a structural and agonistic mimic of a highly homologous epitope from the *Bacteroides* integrase (53). There is also evidence suggesting an association between CD4+ T-cell cross-reactivity against an *E. coli* antigen and the pyruvate dehydrogenase complex (PDC), a major autoantigenic target in human Primary Biliary Chollangitis (PBC) (54). Likewise, human autoimmune gastritis has been associated with T cell cross-reactivity against the *H. pilory* H+, K+–ATPase (55). Furthermore, there is evidence supporting an association between central nervous system (CNS) autoimmunity and cross-reactivity between gut microbial antigens and autoantigenic targets in MS, such as myelin basic protein (MBP) and guanosine diphosphate-L-fucose synthase protein (56–58).

**Table 1:** Antigenic cross-reactivity between autoimmune disease relevant autoantigens and gut/oral microbial T-cell antigens.

Autoimmune Disease	Species	T Cell Response	Autoantigen	Tissue Expression	Peptide	Epitope	Bacteria Species Crossreactivity	Tissue Location	Bacterial Antigen	MHC Restriction	Evidence	Reference
**Rheumatoid Arthritis**	Human	CD4+	N-acetylglucosamine-6-sulfatase (GNS)	Synovial tissue	p222-235	FEPFFMMIATPAPH	*Prevotella spp.*	Gut and oral cavity	Arylsulfatase	ND	Patients show reactivity against autoreactiveand bacterial epitopes	(50)
							*Butyricimonas spp.*	Gut	Commensal peptide			
	Human	CD4+	Filamin A (FLNA)	Synovial tissue	p2446-2460	NPAEFVVNTSNAGAG	*Prevotella spp.*	Gut and oral cavity	Commensal peptide	ND	RA patients show reactivity against autoreactiveand bacterial	(50)
											epitopes	
							*Parabacteroides spp.*	Gut	Commensal peptide			
**Anti-phospholipid Syndrome**	Human	CD4+	Beta-2 glycoprotein I (b2GPI)	Plasma, binds to endothelial cells	p276-290	KVSFFCKNKEKKCSY	*Roseburia intestinalis*	Gut	Commensal peptide	DRB1*04:01	Tetramer reactive CD4+ T cells isolated from blood cross-react with commensal bacteria	(51)
**Systemic Lupus Erythematosus and Sjögren's Syndrome**	Mouse	CD4+	ND	Ubiquitous	p371-381	FLLAVDVSASM	*Capnocytophaga ochracea*	Oral Cavity	Commensal peptide	DRB1*0301	T cells isolated from Ro60-immunized DR3-humanized mice recognize commensal epitope	(52)
**Type 1 Diabetes**	Mouse	CD8+	Islet-specific glucose-6-phosphatase catalytic subunit- related protein (IGRP)	Pancreatic β-cells	p206–214	VYLKTNVFL	*B. vulgatus; B. sp. 4_3_47FAA; B. sp. 9_1_42FAA; B. sp. 3_1_33FAA; and B. dorei 5_1_36/D4*	Gut	Integrase	H-2–K^d^	Bacterial peptide triggers recruitment of low avidity IGRP_206-214-_reactive CD8+ T cells to the gut and protects mice against colitis	(53)
**Primary Biliary Cholangitis**	Human	CD4+	Pyruvate dehydrogenase complex PDC-E2	Ubiquitous	p163-176	GDLLAEIETDKATI	*Escherichia coli*	Gut	Lipoic acid-binding domain of commensal PDC-E2	DRB4 *0101	Specific CD4+ T cell clones isolated from patients crossreact with commensal PDC-E2 protein	(54)
**Autoimmune Gastritis**	Human	CD4+	Gastric enzyme hydrogen potassium adenosine	Gastric mucosa	p621-635	IRVIMVTGDHPITAK	*Helicobacter pylori*	Gut	Histidine kinase	DR	Specific CD4+ T cell clones isolated from autoimmune gastritis patients crossreact with multiple *H. pylori* antigens	(55)
**Autoimmune Gatritis**			triphosphatase (H+,K+–ATPase)		p781-795	NLKKSIAYTLTKNIP			Dimethyl adenosine transferase			
					p46-60	KKEMEINDHQLSVAE			Penicillin-binding protein 2			(55)
					p836-850	KAESDIMHLRPRNPK			LPS biosynthesis protein			
					p181-195	VIRDGDKFQINADQL			Acetate kinase			
					p241-255	CTHESPLETRNIAFF			Phosphoglucosamine mutase			
					p256-270	STMCLEGTAQGLVVN			VirB4 homologue			
					p516-530	VMKGAPERVLERCSS			GidA			
					p621-635	IRVIMVTGDHPITAK			Porphobilinogen deaminase			
**Multiple Sclerosis**	Human	CD4+	Myelin basic protein (MBP)	Central nervous system	p85-99	ENPVVHFFKNIVTPR	*Escherichia coli*	Gut	GTP-binding protein engA	DRB1*1501	Commensal peptide activates and drives EAE inflammation in Ob TCR-DR2b mice	(56)
	Human	CD4+	Guanosine diphosphate-L-fucose synthase ((GDP)-L-fucose synthase)	Central nervous system	p161-175	YGCTFTAVIPTNVFG	*Akkermansia muciniphila*	Gut	Commensal peptide	DRB3*02:02	Identification of guanosine diphosphate (GDP)-L-fucose synthase as an autoantigen that is recognized by cerebrospinal fluid-infiltrating CD4+ T cells from HLA-DRB3- positive patients	(57)
	Mouse	CD4+	Myelin oligodendrocyte glycoprotein (MOG)	Central nervous system	p40-48	YRSPFSRVV	*Lactobacillus reuterI*	Gut	UvrABC system protein A (UvrA)	I-A^b^	*L. reuteri* encodes peptides that potentially mimic MOG. Mice co-colonized with this strain develop more severe experimental autoimmune encephalomyelitis than germ-free or monocolonized mice	(58)

ND, not determined.

Certain pathobionts can promote autoimmune responses through mechanisms other than molecular mimicry. For example, *E. gallinarum*, a pathobiont associated with SLE autoimmunity and autoantibody responses against various SLE and APS-relevant autoantigens, such as RNA, double-stranded DNA and β2GPI, has been detected in gut-distal organs of patients, suggesting a role for bacterial translocation in this process (59). As noted above, the pore-forming toxin of the oral pathobiont *A. actinomycetemcomitans* can citrullinate proteins, leading to neoantigen formation and production of RA-associated autoantibodies (60). Other pathogens can activate autoreactive responses in a non-antigen specific way, by creating an inflammatory environment that promotes bystander lymphocyte activation. For example, in a mouse model of arthritis, SFB antigens induced autoimmune lung inflammation by triggering the formation of autoreactive Th17 cells from naïve T cell precursors co-expressing SFB antigen specific TCRs (61). Notwithstanding these associations, the precise mechanisms and the potential role of these processes in human autoimmune diseases remain unclear.

## Gut Microbiota – Autoimmune Disease Associations

**Figure 4:**
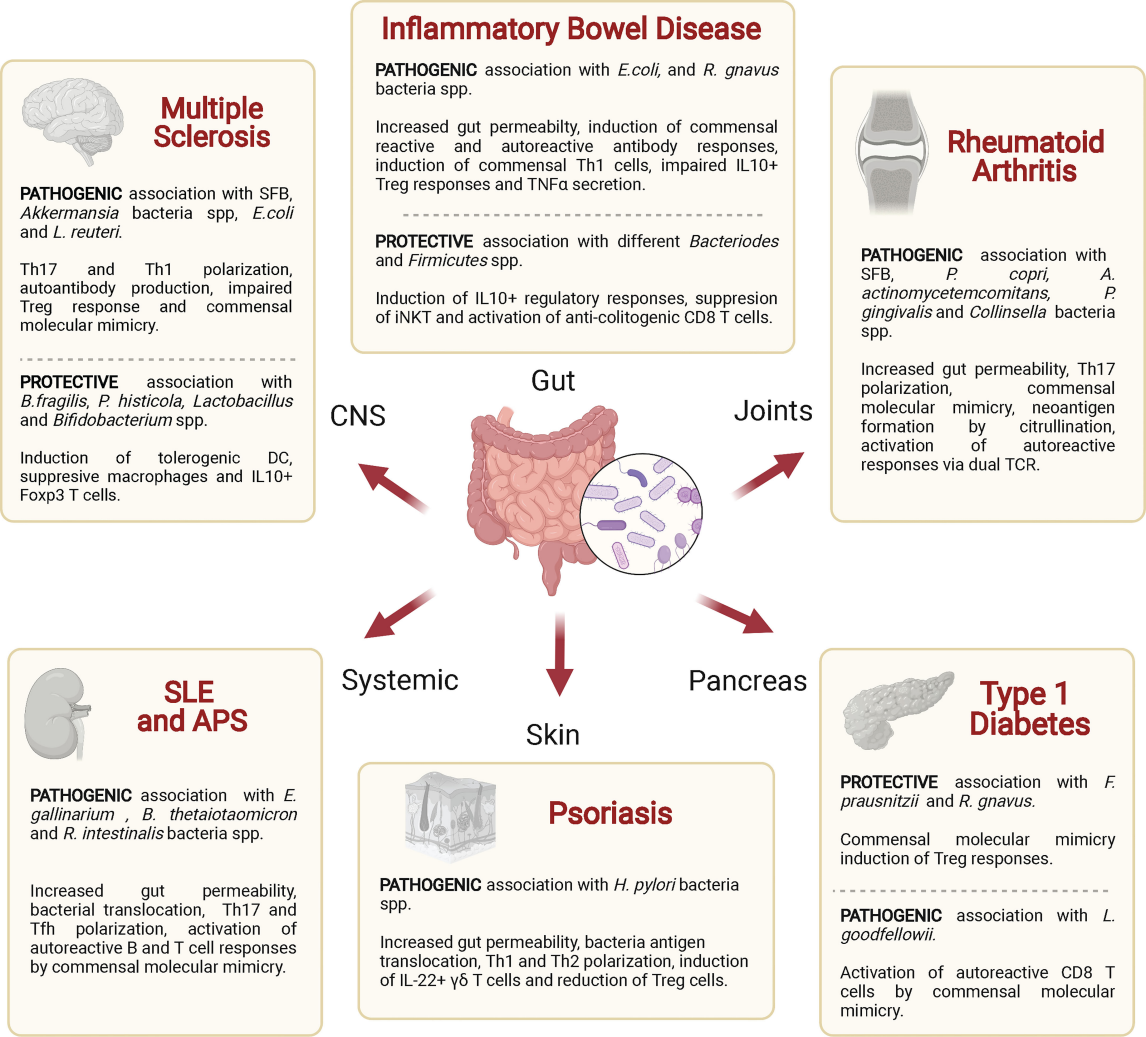
Associations of various autoimmune diseases with commensal bacteria. Specific commensal bacteria may enhance or reduce the host’s susceptibility to specific autoimmune diseases by altering intestinal permeability, polarizing effector or regulatory T cell responses and/or by triggering autoreactive T cell responses *via* antigen mimicry. SFB, segmented filamentous bacteria; DC, dendritic cell; iNKT, natural killer T-cells; Treg, regulatory T cell; TCR, T cell receptor.

Dysbiosis and disruption of the integrity or barrier function of the intestinal epithelium have been associated with various autoimmune diseases. Below, we discuss such associations with a focus on potential mechanisms, including antigenic mimicry (**Table 2** and **Figure 4**).

**Table 2:** Gut and oral bacterial species associated with autoimmune disorders.

Disease Group	Autoimmune Disorder	Bacterial Species	Classification	Tissue Localization	Disease Association	Target Cell Type	Mechanisms [Refs]	Clinical Associations [Refs]	Animal Studies [Refs]
**Gut Axis**	**Inflammatory Bowel Disease (IBD)**	*Roseburia sp, Eubacterium sp.Ruminococcaceae spp., Lachnos piraceae spp., Faecalibacterium prausnitzii*,	Commensal	Gut	Protective	DC and Treg	Bacteria produce SCFA playing a major role in modulation of inflammation, regulation of immune responses and maintenance of barrier integrity in the gut. Also promote expansion of Tregs and skew dendritic cells to prime IL-10 secreting T cells (22, 23, 24).	Decreased levels in IBD patients (62).	CD4+CD25+FoxP3+ T cell numbers increased in the lamina propria of mice treated with *R. hominis*. Treatment with the *R. hominis* bacterium provided protection against dextran sodium sulfate (DSS)-induced colitis (23).
		*Escherichia coli*	Commensal	Gut	Pathogenic	Autoantibodies, Th1 and Tregs	Bacterial antigens induce anti-OmpC antibodies, Th1 cells and impaired CD4+IL-10+ cell responses, promoting intestinal inflammation (63–65).	Increased antibody responses against OpmC were associated with IBD severity. Imparied OmpC-specific IL10-producing CD4+ T cell responses were detected in blood of CD patients (63, 65).	Detected activated Th1 CD4+ T cells against E. coli antigens (64).
		*Ruminococcus gnavus*	Commensal	Gut	Pathogenic	DC	Bacteria secrete a complex glucorhamnan polysaccharide inducing TNFα secretion by DCs through TLR4 signaling (66)	Higher levels of *Ruminococcus gnavus* detected in IBD patients often co-occurring with increased disease activity (67).	Germ-free mice colonized with an unencapsulated strain of *R. gnavus* show increased gut inflammation compared to an encapsulated strain, which stimulates a tolerogenic response *in vivo* (53).
		*B. vulgatus; B. sp. 4_3_47FAA;B. sp. 9_1_42FAA; B. sp. 3_1_33FAA; and B. dorei 5_1_36/D4*	Commensal	Gut	Protective	CD8+	Gut microbial antigen recruits low avidity IGRP206-214/Kd specific CD8+ T cells to the gut, which then promote the killing of gut microbial mimic-loaded dendritic cells, precluding the activation of other T cell effectors (53).	*Bacteroides* integrase reactive CD8+ T cells present in PBMC of type 1 diabetic and Crohn´s disease patients (53).	Low avidity autoreactive IGRP 206-214/Kd-specific CD8+ T cells suppress experimental colitis (53).
		*Bacteriodes fragilis*	Commensal	Gut	Protective	iNKT	Bacteria produce lipid antigens controlling homeostatic iNKT cell proliferation and activation, preserving gut integrity (34).	Higher *B. fragilis* prevalence associates with Crohn's disease exacerbations (69).	Treatment of mice with *Bacteroides fragilis* glycosphingolipids reduces colonic iNKT cell numbers and confers protection against oxazolone-induced colitis (34).
	**Autoimmune Gastritis (AIG)**	*Helicobacter pylori*	Commensal	Gut	Pathogenic	Th1	Bacterial antigens activate pro-inflammatory Th1 CD4+ T cells that recognize H+,K+–adenosine triphosphatase host proteins (55).	Identification of H+,K+–ATPase-specific CD4+ T cells that crossreact with *Helycobacter pylori* in AIG patients (55).	
**Brain/Optical - Gut Axis**	**Multiple Sclerosis (MS**	*Segmented filamentous bacteria (SFB)*	Commensal	Gut	Pathogenic	Th1/Th17 and Treg	Bacteria promote Th17 pro-inflammatory responses (14, 15).	Detected increased levels of *Firmicutes* species in Relapsing vs. Non-relapsing - Remitting MS patients (70).	SFB colonized germ-free mice develop spontaneous EAE (71).
		*Bacteriodes fragilis*	Commensal	Gut	Pathogenic	DC and Treg	Promotes induction of tolerogenic CD103+ DC and expansion of IL-10 FoxP3+ CD39+ CD4 Treg cells trough PSA-TLR2 signaling (31, 32).	Reduced levels of *Bacteroides* species have been detected in a small cohort of pediatric MS patients (72). Disease modifying therapy increased *Bacteriodes* content (73).	EAE protection mediated by oral PSA administration (31, 32).
		*Prevotella histicola*	Commensal	Gut	Protective	DC, Treg and macrophages	Bacteria inhibit pro-inflammatory Th1 and Th17 cells and increase frequencies of CD4+FoxP3+ regulatory T cells, tolerogenic DC and suppressive macrophages (74).	Intestinal Th17 cell frequency is inversely related to the relative abundance of *Prevotella* strains in the human small intestine of MS patients (70).	Inhibits EAE in mice treated with the commensal bacteria (74).
		*Lactobacillus and Bifidobacterium spp.*	Commensal	Gut	Protective	Treg	Bacteria promote Tregs, Th1/Th17 supporting autoreactive responses (75, 76).	In a randomized, double-blind, placebo-controlled trial, oral administration of commensals improved MS disease (77).	Bacterial administration in EAE mice show therapeutic activity (75, 76).
		*Escherichia coli*	Commensal	Gut	Protective/Pathogenic	CD4+ and Treg	*E.coli* Nissle 1917 trigers the recruitment of anti-inflammatory, IL10-producing MOG-specific CD4+ T cells to the CNS (78). Bacterial molecular mimicry (56).		*E.coli* Nissle 1917 reduced the severity of EAE induced by immunization with the MOG 35 - 55 peptide (78). *E.coli* peptide activates and drives EAE in Ob TCR-DR2b mice (56).
		*Akkermansia spp.*	Commensal	Gut	Pathogenic	CD4+ and Treg	Bacteria mimics guanosine diphosphate-L-fucose synthase sequence (79), and also induce impaired Treg responses (48).	Identification of cerebrospinal fluid-infiltrating cells in MS commensal levels also associate with MS disease (48).	*Akkermansia* association with MS was reported in a twin study where mice colonized with patient stool samples harbored Tregs producing lower levels of IL-10 (48).
		*Erysipelotrichaceae family and Lactobacillus reuteri*	Commensal	Gut	Pathogenic	Th17	Bacterial peptides mimic MOG40 - 48 epitope and induces Th17 polarization (58).		Co-colonization with both strains increased EAE severity (58).
	Autoimmune uveitis	Undefined microbiota	Commensal	Gut	Pathogenic	Th1/Th17	Bacteria mimics IRBP autoantigen (80) and also induce Th1/Th17 T cells (81).		R161H mouse model, which expresses the R161 TCR, recognize residues 161–180 of IRBP, a major uveitogenic epitope in B10.RIII mice. These cells can be activated by ommensal microbiota. In addition, germ-free C57BL/6 mice were resistant to experimental autoimmune uveitis (80).
**Endocrine/Exocrine -Gut Axis**	**Primary Biliary Cholangitis (PBC)**	*E. coli*	Commensal	Gut	Pathogenic	CD4+	Bacteria mimic host PDC-E2 molecule (54).	Frequency of PDC-E2 163 - 176 reactive CD4+ T cells is significantly increased in peripheral blood of PBC patients as compared to healthy subjects (82).	CD4+CD25+FoxP3+ T cell numbers increased in the lamina propria of mice treated with *R. hominis* . Treatment with the *R. hominis* bacterium provided protection against dextran sodium sulfate (DSS)-induced colitis (23).
	**Type 1 Diabetes (T1D)**	*Ruminococcus gnavus*	Commensal	Gut	Protective	CD8+ Treg	Bacteria induce CD8+CD122+ regulatory T cells (83).	Compared to healthy individuals, T1D patients have fewer CD8+ Treg cells in association with a lower prevalence of *Ruminococcus* (83).	*Ruminococcus* spp. are more abundant in parasite infected mice and seem to be responsible for the induction of CD8+ Treg cells and suppression of streptozotocin (STZ)-induced diabetes (83).
		*F. prausnitzii*	Commensal	Gut	Protective	DC and Treg	Produce SCFA, playing a major role in modulation of inflammation, regulation of immune responses, and maintenance of barrier integrity in the gut. Also promotes expansion of Tregs and skews dendritic cells to prime IL-10 producing T cells (22, 23, 28).	Decreased levels are detected in children with T1D-associated autoantibody seropositivity (84).	
**Systemic-Gut Axis**	**Systemic Lupus Erythematosus (SLE)**	*Enterococcus gallinarum*	Pathobiont	Gut	Pathogenic	Th1/TFH and Antibodies	Bacteria induce Th17 and TFH responses supporting autoantibody responses (59).	Bacteria was found in liver biopsies of SLE patients, but not in healthy controls (59).	Antibiotic treatment decreases mortality in SLE mice by suppressing growth of *E. gallinarum* in tissues, as well as decreasing pathogenic autoantibodies and autoreactive T cells (59).
		*Bacteroides thetaiotaomicron*	Commensal	Gut	Pathogenic	CD4+ and Antibodies	Bacteria mimic Ro60T, induce specific T and B cell responses (85).	Commensal-reactive T cell clones from SLE patients cross-react with human and bacterial Ro60 protein (85).	Monocolonization of germ-free mice with *B. thetaiotaomicron* triggers T and B cell responses against hRo60 (85).
	**Anti-Phospholipid Syndrome (APS)**	*Roseburia intestinalis*	Commensal	Gut	Pathogenic	CD4+ and Antibodies	Bacteria mimics β2GP1 autoantigen (51).	CD4+ T cells that crossreact with commensal bacterial are detected in blood of APS patients (51).	
	**Rheumatoid Arthritis (RA)**	Segmented filamentous bacteria (SFB)	Commensal	Gut	Pathogenic	Th17 and Antibodies	Bacteria induce Th17 and antibody responses [86].Activation of auto-reactive/SFB epitope cross-reactive T cells expressing two TCRs (61).		Monocolonization with SFB triggers arthritis in germ-free K/BxN mice (86). SFB expand dual T cell receptor (TCR) - expressing Th17 cells recognizing both an SFB epitope and autoantigen in a model of autoimmune arthritis (61).
		*Porphyromonas gingivalis*	Pathobiont	Gut	Pathogenic	Th17 and Antibodies	Bacteria induce specific antibodies and Th17 cell responses by TLR-2 signaling (87, 88). Also increase the antigen repertoire by protein citrullination (89).	Patients with RA have significantly higher titers of anti-*P. gingivalis* antibodies as compared to controls, albeit without any correlation with disease severity (88).	Periodontitis induced by bacteria significantly aggravated the severity of collagen-induced arthritis in mice (87).
		*Aggregatibacter actinomycetemcomitans*	Commensal/Pathobiont	Gut	Pathogenic	Antibodies	Bacteria induce hypercitrullination in host neutrophils via pore- forming LtxA signaling, promoting autoantibody formation (60).	Exposure to Ltxa Aa strains was confirmed in patients with RA and was associated with increased titers of anti-citrullinated protein antibodies and rheumatoid factor (60).	Inhibits EAE in mice treated with the commensal bacteria (74).
		*Prevotella copri*	Commensal	Gut	Pathogenic	Th17	Bacterial molecules mimic RPL23A, and also induce Th17 cell responses (90).	Patients with early RA disease harbored intestinal microbiota dominated by *P. copri* (90).	SKG mice harboring microbiota from RA patients had an increased number of intestinal Th17 cells and developed severe arthritis after zymosan treatment. In addition, naive SKG mouse T cells co-cultured with *P. copri* -challenged dendritic cells produced IL-17 in response to RPL23A antigen and rapidly induced arthritis in mice (90).
		*Collinsella*	Commensal	Gut	Pathogenic	Th17	*Collinsella* correlated strongly with high levels of alpha- aminoadipic acid and asparagine as well as production of the proinflammatory cytokine IL-17A in RA patients (91).		A role for *Collinsella* in altering gut permeability and disease severity was confirmed in experimental arthritis (91).
**Skin-Gut Axis**	**Psoriasis**	*Helicobacter pylori*	Commensal/Pathobiont	Gut	Pathogenic	Th1/Th2 and Treg	Bacteria induce intestinal permeability, increasing antigen translocation across gut mucosa (92). The enterotoxin secreted by *H. pylori* polarizes Th1/Th2 responses and also decreases Treg cell frequencies (93).	*H. pylori* infection associates with progression of psoriatic disease (94).	

SCFA, short-chain fatty acids; Treg, regulatory T cell; OpmC, outer membrane porine C; TLR4, toll-like receptor 4; PBMC, peripheral blood mononuclear cell; iNKT, invariant natural killer T-cells; EAE, experimental autoimmune encephalomyelitis; DC, dendritic cells; PSA, polysaccharides A; CNS, central nervous system; MOG, myelin oligodendrocyte glycoprotein; IRBP, interphotoreceptor retinoid-binding protein; TCR, T cell receptor; PDC-E2, pyruvate dehydrogenase complex E2; Ro60T, RNA binding protein; β2GP1, Beta-2 glycoprotein I; RPL23A, arthritis-related autoantigen ribosomal protein L23a; Spp., specie; LtxA, toxin leukotoxin A.

### Inflammatory Bowel Disease

CD is a form of IBD that can affect any part of the gastrointestinal tract, but predominantly targets the terminal ileum and colon. Ulcerative colitis (UC) is another form of IBD which only targets the colon. Despite the fact that the incidence and prevalence of IBD are increasing worldwide and are appearing earlier in life, the etiology and pathogenesis of CD and UC remain ill-defined (95). Although there is an important underlying genetic component (96), disease development requires an environmental trigger (97). Many studies have provided evidence for the loss of gut microbial tolerance in human IBD (98), including the development of B and T cell responses against gut microbial antigens and autoantigens (99–106).

Whereas CD has been generally associated with increased Th17- and Th1-type responses, UC is primarily associated with Th17- and Th2-type responses (107). In CD patients, Th1-associated transcription factors such as STAT4 and T-bet, and cytokine receptors such as IL-12Rβ2 are highly expressed in the lamina propria of the inflamed gut (108). Likewise, the development of CD-like ileitis in SAMP1/YitFc and TnfΔARE mice has been associated with Th1-driven inflammation (109, 110). Indeed, Th1-type cells appear to be necessary for gut inflammation since immune cells from *Ifng*^–/–^, *Tbx21*^−/−^, and *Stat4*^−/−^ donors cannot transfer intestinal inflammation into immunocompromised hosts (111–113).

There is also strong evidence for the contribution of Th17-type responses to human IBD. Mucosal biopsies from both CD and UC patients contain increased levels of Th17 cell-derived cytokines (114, 115). In addition, human IBD is associated with genetic polymorphisms at loci encoding Th17 pathway components (e.g., *IL6ST, JAK2, STAT3, RORC, IL23R, CCR6*) (116). In agreement with these observations, *Stat3*^–/–^ and *Rorc*^–/–^ mice are resistant to experimental colitis (117, 118). However, since Th17-type cells are known to contribute to the maintenance of normal gut microbial homeostasis, it seems likely that the pathogenic Th17-like cells that contribute to IBD are a different subset (119, 120). Th17-type cells, like other Th cell subsets, are plastic, and the associations between Th17-type cells and both IBD and other autoimmune disorders appear to be mediated by Th17-like cells co-expressing IFNγ and IL-17A (121, 122). In mice, pathogenic Th17-type cells express high levels of the IL-23 receptor (123, 124), and the *IL23R* gene is strongly associated with human IBD (116). Interestingly, a subset of intestinal human memory CCR6^+^CXCR3^+^ T cells co-expressing Th17 and Th1 markers from CD patients express the Multidrug Resistant Mutation MDR1, a plasma membrane drug efflux pump (125) that is encoded in a gene strongly associated with IBD (*ABCB1*) (126).

Although the role of Th2-type cells in the pathogenesis of IBD remains unclear, studies in both humans and mice support their involvement. For example, biopsies of UC patients contain increased levels of IL-4 (127) and sera from both UC patients and mice with oxazolone-induced colitis contain elevated levels of IgE, an IL-4-regulated immunoglobulin isotype (128, 129). Likewise, development of ileitis in SAMP1/YitFc mice, and colitis in the TNBS-induced model are associated with Th2-type responses (130, 131).

The onset of IBD has been linked to both microbial dysbiosis and disruption of gut epithelial permeability (132). Decreased abundance on Firmicutes bacteria belonging to the *Ruminococcaceae* spp., *Lachnospiraceae spp, F. prausnitzii* and *Roseburia* spp. families is a signature of microbial dysbiosis in IBD (62, 133). Since these bacteria are butyrate producers, this association may be driven by altered (reduced) induction of iTreg cells in the gut (134, 135). In agreement with this, polymorphisms in genes coding for the immunoregulatory cytokine IL-10 (*IL10*) or subunits of the IL-10 receptor (*IL10RA, IL10RB*) are strongly associated with human IBD, particularly with early onset forms of colitis (136). Increased prevalence of pro-inflammatory commensals is yet another mechanism through which dysbiosis may contribute to the pathogenesis of IBD. For example, gut inflammation in patients and murine models has been associated with increased prevalence of *R. gnavus* (67, 68), which triggers the production of inflammatory cytokines (e.g. TNFα) by DCs *via* polysaccharide signaling (66). The role of other commensals is less clear. For example, although *B. fragilis* metabolites promote barrier integrity, the prevalence of this bacteria has been associated with disease exacerbation in CD patients (69), suggesting context-dependent effects.

Increased antibody responses against various gut microbial antigens have been described in IBD. For example, CD patients have increased serum titers of antibodies against the *E. coli* membrane porin C (OmpC), yeast *S. cerevisiae* mannose epitopes (ASCA) and bacterial flagellins (CBir) (105, 106), and both the presence and titers of these antibodies are associated with disease severity (63). CD4+ T cell responses against some of these gut microbial antigens have also been associated with IBD. For example, activated OmpC-specific CD4+ T cells are colitogenic in mice (64), and OmpC-specific CD4+ T-cells have been detected in the peripheral blood of IBD patients (65). There is also evidence for a role of flagellin-specific CD4+ T cells; increased frequencies of flagellin-specific CD4+ T cells with an activated, gut homing phenotype were detected in CD and UC patients versus controls. Furthermore, CD, albeit not UC, is associated with increased serum levels of anti-flagellin IgG and IgA antibodies (137).

Many studies have provided evidence for the contribution of an autoimmune component in the maintenance of chronic intestinal inflammation. Most UC, and to a lesser extent CD patients develop peri-nuclear anti-neutrophil cytoplasmic antibodies (pANCA) (99, 138). These antibodies cross-react with the OmpC protein, suggesting a possible role for B cell autoreactivity and gut microbial antigenic cross-reactivity in the pathogenesis of IBD (139). Likewise, IBD has been associated with autoantibody responses against Glycoprotein 2 (GP2) (100, 101), a receptor for bacterial adhesin FimH that is upregulated in the gut epithelium of patients (100). Of note, high levels of anti-GP2 IgA antibodies have been described in pediatric IBD patients (101). An increased prevalence of autoantibodies against FAM84A, a neuronal sensory protein expressed in the gastrointestinal tract, has also been associated with IBD (102).

The contribution of autoreactive T-cell responses to IBD is much less clear. Some studies have reported the ability of commensal bacteria to activate colitogenic T-cells or autoreactive T cells. A *Citrobacter* infection in ovalbumin specific TCR transgenic mice triggered the apoptotic cell death of infected colonic epithelial cells, promoting intestinal inflammation *via* the activation of autoreactive Th17 CD4+ T cells (45). Importantly, there is also evidence for protective autoreactive T cell responses in IBD. Specifically, a *Bacteroides* integrase epitope was shown to induce the recruitment of a highly prevalent low avidity IGRP_206-214_ specific CD8+ T cell subset to the gut, affording the mice protection against experimental colitis (53).

The above observations suggest that the relative contribution of autoimmune vs. non-autoimmune phenomena to UC and CD is different. Thus, whereas autoreactivity against colonic epithelial cells may play a role in UC, immune reactivity against the intestinal flora is primarily a feature of CD. Multiple environmental factors, genetic determinants as well as the specific contribution of commensal bacteria to dysbiosis could bias the inflammatory response and the disease phenotype in each of these two inflammatory bowel diseases.

### Multiple Sclerosis

MS is a CNS-specific autoimmune disease that is largely driven by Th17-type cells and is characterized by CNS inflammation, demyelination, and progressive neurodegeneration (140). Most patients suffer a relapsing-remitting form of disease (RR-MS). Although the etiology of MS is complex and incompletely understood, both genetic and environmental factors clearly play a role (141, 142).

Several studies have provided evidence for associations between dysbiosis and MS, such as increases in the prevalence of *Methanobrevibacter* (Archaea) and *Akkermansia* (143) or firmicutes (70), as well as a reduction in the prevalence of *Butyricimonas* (143).

In experimental autoimmune encephalomyelitis (EAE), a mouse model of MS, induction of autoreactive B cell responses against myelin oligodendrocyte glycoprotein (MOG) requires the presence of the microbiota (144). In addition, induction of EAE in germ-free mice was associated with reduced levels of IL17 and IFNγ in both the intestine and spinal cord as well as increased levels of Treg cells. Interestingly, colonization of these germ-free mice with SFB restored EAE susceptibility, implicating the microbiota on the development of encephalitogenic Th17 responses (71). More recently, two different bacteria from the *Erysipelotrichaceae* family and *L. reuteri* have been associated with the severity of EAE *via* effects on Th17 cells and MOG molecular mimicry, respectively (58). Another study reported defective production of IL-10 by Treg cells from mice colonized with fecal samples from MS patients, suggesting that MS patients harbor a specific repertoire of commensals that favor CNS autoimmunity (48).

Cross-reactive T cell responses against gut microbial antigens have also been described in MS. In a recent study, cerebrospinal fluid (CSF)-infiltrating T cells specific for GDP-L-fucose synthase cross-reacted with *Akkermansia* antigens (79). Furthermore, GDP-L specific clones recognized the myelin basic protein (MBP) epitope MBP_83-99_, suggesting that T cell cross-reactivity between gut microbial and CNS autoantigens could act as a trigger of CNS inflammation.

Other studies reported protective effects of certain commensal bacteria against CNS autoimmunity. Administration of *B. fragilis* PSA has been shown to protect mice against CNS autoimmune inflammation by promoting the expansion of Foxp3+ Tregs expressing CD39 (31) and by inducing tolerogenic DCs (32). Likewise, administration of *P. histicola* resulted in reduced frequencies pro-inflammatory Th1 and Th17 cells and increased frequencies of FoxP3+ Treg cells, tolerogenic DCs and suppressive macrophages (74). Other species, such as *Bifidobacterium* and *Lactobacillus* have been shown to protect mice against EAE by promoting Treg cell responses and reducing Th1- and Th17-type responses (75, 76). The *E. coli* Nissle 1917 strain was also shown to suppress CNS inflammation by promoting the formation of IL-10-producing autoreactive Treg cells (78).

Similar observations have been reported in humans. A study in a small cohort of pediatric MS patients reported a reduced prevalence of *Bacteroides* (72). In addition, increases in the *Bacteroides* content of the gut microbiota of RRMS patients with commensal modifying therapies was associated with disease-protective effects (73). Likewise, a reduced prevalence of *Prevotella* strains has been associated with increased frequencies of Th17 cells and disease activity in MS patients (70), suggesting a potential protective role for these bacteria against CNS inflammation. In addition, gut microbiota from MS patients imprinted defective IL-10 responses in fecal transplanted host mice, promoting the development of spontaneous EAE (48). In a recent human clinical trial, oral delivery of *Lactobacillus and Bifidobacterium spp* ameliorated MS symptoms (77).

### Systemic Lupus Erythematosus

SLE is a systemic (multi-organ) autoimmune disease characterized by development of autoantibody responses against nucleic acids, histones and ribonucleoproteins, leading to the formation and deposition of pathogenic immune complexes in various organs, including the kidney. Th17 polarization and higher frequencies of TFH cells have been described in the peripheral blood of SLE patients (145), consistent with the extensive autoantibody response underlying disease pathogenesis. The etiology of SLE, as is also the case for most other autoimmune diseases, remains unclear. There is an important genetic component that, although necessary, is insufficient for disease development (146). Environmental cues, such as infectious agents, are suspected to play a role as triggers of disease development in individuals at risk.

Recent evidence points to the microbiota as another potential contributing factor to the development of SLE (147, 148). Disruption of the barrier function of the gut, leading to translocation of commensal bacteria and pathobionts is one of the possible mechanisms underlying this association. For example, *E. gallinarum* was detected in the liver of SLE patients (as well as in the liver of patients with autoimmune hepatitis) but not in the liver of healthy controls (59). In a murine model of SLE, antibiotic treatment reduced mortality and decreased the production of pathogenic autoantibodies and autoreactive T cells, in part by suppressing the growth of *E. gallinarum* in tissues (59).

The composition of the early gut microbiota in mice also appears to have an impact on the development of anti-nuclear autoantibodies (149). SLE patients develop autoantibodies against the evolutionarily conserved RNA binding protein Ro60 (150, 151). Multiple gut commensals, such as *B. thetaiotaomicron*, express Ro60 orthologues with high sequence homology to human Ro60. Furthermore, colonization of germ-free mice with *B. thetaiotaomicron* led to the development of T and B cell reactivity against Ro60, as well as to glomerular immune complex deposition mimicking lupus nephritis (85). The same study reported that bacterial Ro60-specific T cell clones isolated from SLE patients cross-reacted with the human orthologue. Together, these data provided evidence for gut microbial molecular mimicry as a potential contributor to the development of SLE.

### Type 1 Diabetes

T1D is a multifactorial autoimmune disorder characterized by immune-mediated destruction of the pancreatic β-cells, in which numerous genetic elements and putative environmental triggers play a role. Several different alterations of gut microbial health have been associated with T1D in both animal models and humans (152–154), including alterations of intestinal permeability (155, 156), as well as loss of gut microbial diversity before the onset of disease (157). Pro-diabetogenic, oral antibiotic-induced gut dysbiosis in NOD mice has been associated with impaired enteric Th17/Treg responses (158). More recently, *R. gnavus* has been suggested to protect mice against streptozotocin (STZ)-induced diabetes, as well as to promote the development of anti-diabetogenic CD8+CD122+ Treg cells in T1D patients (83).

Gut microbial molecular mimicry has also been implicated as a possible mechanism of autoreactive T-cell activation in the pathogenesis of T1D. A protein from *L. goodfellowii* was suggested to function as a structural mimic of the murine diabetogenic IGRP_206‐214_ epitope, as it could promote the activation of cognate TCR-transgenic CD8+ T-cells *in vitro* (159). In another study, however, metagenomic sequencing of the gut microbiota failed to verify the presence of *L. goodfellowii* in the gut microbiota of both mice and patients (53). Most importantly, the latter study identified the *Bacteroides* integrase, an abundant gut microbial antigen, as a true structural and functional mimic of IGRP_206‐214_ (53). However, experiments in mono-colonized germ-free mice indicated that this gut microbial epitope promotes the recruitment and activation of anti-colitogenic, low avidity IGRP_206-214_-specific CD8+ T-cells, rather than the activation of their diabetogenic high-avidity counterparts (53).

Gut microbial metabolites have also been implicated in the immunopathogenesis of T1D. Increased prevalence of *Bacteroides* species as well as deficiencies in bacteria that produce SCFAs have been described in T1D patients (84, 160). For example, children with T1D-associated autoantibody seropositivity have a reduction in the abundance of the butyrate producer *F. prausnitzii* (84). Another multicenter study of 783 children showed that the microbiota of healthy children is enriched in SCFA-producers, without obvious associations with any taxa, suggesting that microbial function rather than composition might contribute to T1D development (161). In agreement with these observations, NOD mice fed with diets promoting gut microbial production of acetate and butyrate were almost completely protected from T1D *via* SCFA-mediated immunomodulation (162).

### Rheumatoid Arthritis

RA is an organ-specific autoimmune disease that is characterized by chronic inflammation and progressive destruction of the joint tissues by arthritogenic T cells and autoantibodies. Although the pathogenesis of RA remains incompletely defined, both genetic and environmental factors, including alterations in the gut microbiota, have been implicated in its development. As is the case for the other autoimmune diseases discussed above, alterations in intestinal permeability (163) and gut microbial composition (164) have been found to predate the onset of disease in RA. Commensal bacteria such as *Collinsella* have been associated with increased gut permeability and disease severity in both an experimental model of arthritis and in human RA. In RA patients, for example, pro-arthritogenic IL-17A responses in a subset of RA patients were associated with an increased prevalence of *Collinsella* (91). In another study, colonization of germ-free mice with SFB bacteria was sufficient to induce arthritogenic Th17 responses (86).

Other studies have suggested a role for molecular mimicry as a trigger of arthritogenic autoimmune responses in both animal models and patients. An early work reported the presence of immunoreactivity against an *E. coli* epitope, QKRAA, in the synovial fluid of patients as compared to controls (165). A more recent study found that autoreactive CD4+ T cells against the autoantigens Filamin A (FLNA) and N-acetylglucosamine-6-sulfatase (GNS) cross-react with similar sequences found in *Prevotella*, *Butyricimonas* and *Parabacteroides* species (50). Furthermore, increased prevalence of *Prevotella* species, such as *P. copri* were detected in patients with new-onset RA (166). In mice, *Prevotella* has also been proposed to contribute to RA development, in this case by both, activating autoreactive T cells specific for the arthritis-relevant autoantigen Ribosomal Protein L23a (RPL23A), and by inducing pro-inflammatory Th17 responses (90).

The oral microbiota has also been implicated in RA. Periodontitis induced by *P. gingivalis*, an established oral pathobiont linked to this condition, has been associated with the exacerbation of autoimmune arthritis, presumably by inducing pathogenic Th17 responses *via* TLR2- and IL-1-signalling (87). Of interest, *P. gingivalis* has been found to contribute also to the generation of citrullinated proteins (antigenic targets of RA) in the oral cavity of RA patients (89, 167), suggesting a potential link between immune responses against these post-translationally modified oral proteins and downstream joint inflammation (168). This property has also been documented for another RA-associated oral pathobiont, *A. actinomycetemcomitans* (60).

### Skin Autoimmunity

Psoriasis is a prevalent autoimmune disease characterized by keratinocyte hyperproliferation and skin inflammation, where both genetic and environmental factors also play a role (169). Psoriatic skin lesions are associated with dermal and epidermal infiltration of leukocytes, triggered and maintained by T lymphocytes (170, 171). Most of the T cells that infiltrate the psoriatic dermis are CD4+, whereas those that infiltrate the epidermis are primarily CD8+ (171, 172). Different clinical phenotypes have been associated with the presence of bacterial skin commensals capable of inducing local pro-inflammatory Th17 responses (173). However, multiple studies have also underscored the importance of the gut-skin axis on cutaneous autoimmunity. Gut dysbiosis induced by oral antibiotic treatment in neonatal mice promoted the development of psoriasis by increasing the frequency of cutaneous IL-22 producing γδ+T cells (174). In addition, induction of experimental psoriasis *via* imiquimod exposure is blunted in germ free or antibiotic treated mice, in association with a reduction in Th17 cells (175, 176). In particular, *Helicobacter pylori* infection has been associated with psoriasis (94), potentially *via* both local and systemic effects of the inflammatory response (177, 178), such as increased permeability of the gastric mucosa to food antigens, among others (92). In addition, the *H. pylori* enterotoxin binds to the T cell receptor and induces the expression of T cell skin homing receptors (179, 180).

Vitiligo is another T cell-dependent autoimmune disorder of the skin characterized by skin depigmentation due to immune mediated killing of melanocytes (181). Recently, in a murine model of vitiligo harboring tyrosinase-reactive T cells, oral ampicillin treatment decreased disease severity, suggesting that the gut microbiota may also play a role in this disease (182).

## Concluding Remarks

The specific role that gut microbes, metabolites or gut microbial antigens play in the pathogenesis of autoimmune disease is complex and remain ill-defined. There are clear associations between gut dysbiosis and increased intestinal permeability with several autoimmune phenomena. However, whether these abnormalities contribute to, or are merely a bystander effect of disease progression remains to be addressed. Although experiments in gnotobiotic mice have provided useful information in this regard, it is unclear to what extent the presence of an altered immune system in the germ-free mice that were used in these studies might have affected the study outcome. Bacterial translocation due to gut barrier disruption can lead to increased presentation of gut microbial antigens to the immune system. As a result, activation of autoreactive T and B cells by cross-reactive gut microbial antigens remains a potential mechanism, but the evidence providing direct links between gut microbial antigen cross-reactivity and pathogenic autoimmunity remain largely circumstantial in nature. A more extensive use of reductionist systems of autoimmunity (e.g., TCR-transgenic mice), coupled to mono-colonization of germ-free mice with wild-type and mutant gut microbial species (53) should help address this knowledge gap. The links between the effects of gut microbe-derived metabolites (e.g., SCFA) on the gut-associated lymphoid tissue and autoimmune disease are compelling and intriguing but will need to be integrated into the poorly understood sequence of events underlying the corresponding autoimmune diseases, including their genetic underpinnings.

Notwithstanding these limitations, the studies summarized herein strongly support multifaceted roles for the gut microbiota on autoimmune disease susceptibility or resistance. A precise understanding of each of the many potential mechanisms through which commensal bacteria can promote or protect against autoimmune disorders will help conceptualize novel therapeutic applications in this area.

## Author Contributions

NG and PS wrote the manuscript. Both authors contributed to the article and approved the submitted version.

## Conflict of Interest

PS is scientific founder of Parvus Therapeutics Inc. and has a financial interest in the company.

The remaining authors declare that the research was conducted in the absence of any commercial or financial relationships that could be construed as a potential conflict of interest.

## Publisher’s Note

All claims expressed in this article are solely those of the authors and do not necessarily represent those of their affiliated organizations, or those of the publisher, the editors and the reviewers. Any product that may be evaluated in this article, or claim that may be made by its manufacturer, is not guaranteed or endorsed by the publisher.
